# Distress, anxiety and depression in patients with brain metastases before and after radiotherapy

**DOI:** 10.1186/1471-2407-14-731

**Published:** 2014-09-30

**Authors:** Marie-Christine Cordes, Angela Scherwath, Tahera Ahmad, Ansa Maer Cole, Gundula Ernst, Karina Oppitz, Heinrich Lanfermann, Michael Bremer, Diana Steinmann

**Affiliations:** Department of Radiation Oncology, Medical School Hannover, Carl-Neuberg-Str. 1, 30625 Hannover, Germany; Department and Outpatient Clinic of Medical Psychology, University Medical Centre Hamburg-Eppendorf, Hamburg, Germany; Department of Radiation Oncology, Hospital Braunschweig, Braunschweig, Germany; Department of Medical Psychology, Medical School, Hannover, Germany; Institute for Neuroradiology, Medical School Hanover, Hanover, Germany

**Keywords:** Brain metastases, Distress thermometer, HADS, Whole-brain radiotherapy, hypofractionated stereotactic radiotherapy

## Abstract

**Background:**

Many patients with cancer suffer from distress, anxiety and depression. However, studies on patients with brain metastases are lacking. In this exploratory study we prospectively assessed distress, anxiety and depression in patients with brain metastases from different solid primary tumour treated with radiotherapy to the brain.

**Methods:**

Patients were recruited between May 2008 and December 2010. Distress, anxiety and depression were subjectively evaluated before radiotherapy, 6 weeks, 3 months and 6 months after radiotherapy using the validated National Comprehensive Cancer Network Distress Thermometer (DT) and the Hospital Anxiety and Depression Scale (HADS). The treatment group consisted of adult patients (n = 67) with brain metastases who were treated with whole-brain radiotherapy (n = 40) or hypofractionated stereotactic radiotherapy (n = 27). The control group comprised of patients (n = 32) diagnosed with breast cancer without cranial involvement who received adjuvant whole breast radiotherapy. Forty-six patients (24 in the treatment group) completed the study after six months.

**Results:**

Before radiotherapy, the treatment group experienced higher distress than the control group (p = 0.029). Using a cut-off ≥5, 70% of the treatment group were suffering from significant distress (66% of the control group). No significant time-by-group interaction on distress, anxiety and depression was observed. At all time points, a high proportion of patients reported psychological stress which featured more prominently than most of the somatic problems. Global distress correlated strongly with the Hospital Anxiety score before radiotherapy, but only moderately or weakly with both HADS scores after radiotherapy with the weakest association 6 months after radiotherapy.

**Conclusion:**

In conclusion, the course of distress, anxiety and depression does not differ significantly between patients with brain metastases and breast cancer patients without cranial involvement. This finding suggests that both groups need similar psychological support during their treatment. Both screening instruments should be used as they cover different facets of distress.

## Background

The incidence of brain metastases (BM) in adults who suffer from cancer is more than 25% [1]. BM most frequently originate from cancers of the lung, breast, colon, kidney as well as from cancers of unknown primary (CUP) and melanomas [1]. Because of a median survival time between 3 and 6 months the prognosis of BM is poor [1]. The therapy of BM depends on the size and number of metastases and the overall prognosis [2]. In cases with multiple BM whole brain radiation therapy (WBRT) is administered [2]. Neurosurgery and/or radiosurgery or hypofractionated stereotactic radiotherapy (hfSRT) is indicated in cases with a limited number of lesions [2].

In general, many patients experience a multitude of physical, psychological and psychosomatic symptoms after being diagnosed with cancer. This often results in a deterioration of the physical and psychosocial condition of these patients [3]. The overall prognosis of adult brain tumours is often very poor so that a lot of patients and their spouses found the first diagnosis of a brain tumour very distressing [4]. Depending on the cut-off score used, between 48% and 73% of the patients with brain tumours experience clinically significant distress during the early treatment phase [4, 5]. While somatic symptoms were mentioned by these patients, emotional strain was identified as the major cause of distress [5]. Study results show that 30% of patients being treated for a brain tumour suffer from anxiety and 17% suffer from depression [6].

The Hospital Anxiety and Depression Scale (HADS) [7] and the National Comprehensive Cancer Network’s (NCCN) Distress Thermometer (DT), which has been validated in patients with intracranial tumours [8], are widely used instruments to measure distress in patients with brain tumours [4–6, 9] as well as other cancer populations [10]. As the DT alone does not provide sufficient insight into the reasons for distress, an additional problem list can give information about the sources of distress [11]. The HADS identifies symptoms of anxiety and depression in patients with somatic disorders, but previous studies could not show that brief screening tools like the DT show inferior results [5, 10]. DT was found to be significantly correlated with anxiety and depression levels [11].

To the best of our knowledge, all of the mentioned studies analysed data from patients with brain tissue-specific tumours without including patients with BM. Because of both the high prevalence of BM and the additional psychosocial burden of experiencing a secondary cancer after treatment for the primary cancer disease, it is also important to explore levels of distress in this patient group. Our study aims to investigate distress, anxiety and depression in patients with BM before and after radiotherapy (RT) with the aid of the DT and the HADS. Furthermore, we want to compare this data with a control group (CG) consisting of patients with breast cancer without any metastases who were treated with adjuvant whole breast RT. To determine the degree of overlap of the constructs measured at each time point, correlations between the study instruments will be calculated.

## Methods

### Patients, recruitment and inclusion criteria

Patients with newly diagnosed BM from any solid primary tumour made up the treatment group (TG). They were recruited between May 2008 and December 2010 in the Department of Radiation Oncology at the Medical School Hannover. These patients were treated with WBRT or hfSRT. Seventeen patients of the TG had been treated with up-front neurosurgery of the BM. Exclusion criteria for the TG were chemotherapy during the time of irradiation or prior RT of the brain.

The CG comprised of breast cancer patients who were recruited from March 2010 to December 2010 in the Department of Radiation Oncology at the Medical School Hannover. After breast-conserving surgery or mastectomy, these patients received adjuvant RT of the breast or chest wall with or without RT of the regional lymph nodes. We chose these patients as controls because breast cancer patients are one of the main groups of patients with BM. In addition to that, due to the high frequency of this cancer, breast cancer patients give us the possibility of efficient recruitment and of comparison with data in literature.

All patients included in this study had to fulfill the following criteria: age ≥ 18 years, Karnofsky performance score (KPS) ≥ 70, sufficient comprehension, sufficient understanding of the German language and to be without major psychological impairments. In addition to that, they were obliged to submit an informed consent in writing before inclusion into the study and were free to drop out of the study at any time. The study was approved by the local ethics committee of the Medical School Hannover.

### Radiotherapy technique

All patients were informed about the different therapeutic options before RT. Patients belonging to the TG were treated with WBRT or hfSRT only (without WBRT) as indicated. Patients with multiple BM were treated with WBRT while hfSRT was applied to patients with limited BM (1–3) with different fractionation schedules depending on the size, number and location of the BM. During RT, patients wore a thermoplastic head mask which served to immobilize the head. WBRT was applied with 30 Gy in 10 fractions with opposing lateral fields. HfSRT was achieved by applying the radiation via four to six beams up to 30 Gy in 5 fractions or up to 40 Gy in 10 fractions. Before applying hfSRT, an axial MRI scan and a helical planning CT scan of two mm thickness images were conducted. After this, the CT planning scan was fused with the MRI scan. This improved the preciseness of the target volume definition due to increased visibleness of the BM in the T1 weighted contrast.

The CG was treated with surgical resection of the breast cancer followed by adjuvant RT to the breast or chest wall using a 3D planning procedure. If indicated, RT of the periclavicular lymph nodes and / or a boost to the tumour region was also applied. For radiation, patients were rested on their back on commercial breast boards. Tangential fields were used for whole breast or chest wall radiotherapy up to 50 Gy in 25–28 fractions. RT techniques are shown in Table 1.

**Table 1:** **Patient and treatment characteristics**

	Patients with brain metastases	Patients with breast cancer
	n (%)	n (%)
Patients	67 (100)	32 (100)
Gender		
Male	34 (50.7)	0 (0)
Female	33 (49.3)	32 (100)
Age (Years)		
Median (range)	59.85 (30–73)	57 (39–74)
Family status		
single	9 (13.4)	7 (21.9)
married	42 (62.7)	18 (56.3)
divorced	7 (10.4)	3 (9.4)
widowed	6 (8.9)	4 (12.5)
missing values	3 (4.5)	0 (0)
Educational level		
basic education^l^	1 (1.5)	0 (0)
intermediate education^l^	23 (34.3)	10 (31.3)
higher education^l^	20 (29.9)	12 (37.5)
A level^m^	4 (6.0)	5 (15.6)
High School/ university^h^	11(16.4)	4 (12.5)
Other	1 (1.5)	0 (0)
missing values	7 (10.4)	1 (3.1)
Professional situation		
in education	1 (1.5)	1 (3.1)
employed	15 (22.4)	12 (37.5)
unemployed	3 (4.5)	0 (0)
housewife	6 (9.0)	6 (18.8)
pensioner	31 (46.3)	13 (40.6)
Other	3 (4.5)	0 (0)
missing values	8 (11.9)	0 (0)
Primary		
NSCLL	35 (52.2)	0 (0)
SCLC	8 (11.9)	0(0)
Breast	9 (13.4)	32 (100)
Melanoma	5 (7.5)	0 (0)
RCC	3 (4.5)	0 (0)
colorectal	1 (1.5)	0 (0)
Other	6 (9.0)	0 (0)
RT technique	Whole-Brain RT	Whole-Breast RT
	30 Gy (10x3 Gy): 33 (49)	50 Gy (25-28x1.8-2.0 Gy): 25 (78)
	Other: 7 (10.5)	
	40.05 Gy (15x2.67 Gy): 5 (19)	
	Fractioned stereotactic RT	Other: 2 (4)
	40 Gy (10x4 Gy): 8 (12)	With additional boost
	35 Gy (7x5Gy): 12 (18)	(5x1.8-2.0 Gy): 13(41)
	30 Gy (5x6Gy): 4 (6)	
	Other: 3 (4.5)	
Barthel-Index		
Median (range)	100 (60–100)	100 (100)
60-80	7 (11.3)	
85-94	2 (3.2)	
95-100	53 (85.5)	32 (100)
Up-front chemotherapy	43 (64.2)	18 (56.3)
Up-front surgery	17 (25.4)	32 (100)
RPA		
I	23 (34.3)	-
II	43 (64.2)	-
III	1 (1.5)	-
Number of BM		
1-3	37 (55.2)	-
>4	29 (43.3)	-
Unkown	1 (1.5)	-

*Abbreviations:* h = high education, l = low education, m = middle education, NSCLL = non small cell lung cancer, RCC = renal cell carcinoma, SCLC = small cell lung cancer.

### Study design and procedures

This study is a prospective, longitudinal, single-centre study. Distress, anxiety and depression were evaluated using the DT and the HADS at four different points in time: before RT, 6 weeks, 3 months and 6 months after RT. At each point in time, the Barthel Index and the KPS were recorded for the TG. If the questionnaire was not returned the patient received one reminder. Due to ethical reasons, we decided not to send further reminders in case the medical condition of the patient was poor.

Demographic information on gender, age, marital status, level of education and employment status was collected before the start of RT (Table 1). Additionially, Recursive Partitioning Analysis (RPA) classification of the Radiation Therapy Oncology Group (RTOG) was used to divide patients into three prognostic groups depending on age, KPS, primary tumour classification and presence of extra cranial metastases [12].

### Psychological instruments

Firstly, we used the NCCN DT developed by the NCCN as a screening tool. It consists of a visual analogue scale ranging from 0–10 points and like in a thermometer increasing numerical values signify increasing distress levels [3, 11]. Patients have to circle the number on the DT which describes their level of distress over the last week [11]. According to the NCCN Distress Management Guidelines [13] and recommendations of the authors of the German version [11], a score of 5 or greater signifies a distress level where the patient needs support. In addition, the DT includes a problem list with five domains (practical problems, family issues, emotional stress, spiritual concerns and physical ailments). The version we used [11] consists of 34 dichotomous items indicative of the presence of a problem within the last 7 days (yes or no). In addition, one item asks for other problems not included in the problem list. The problem list cites possible reasons for the distress [11].

The second instrument that we applied was the German version of the HADS, which was developed to screen for symptoms of anxiety and depression in patients with physical illnesses [14] and includes 14 items (seven for each anxiety and depression). Each item is scored from 0 to 3 (total 21 points). Higher values signify greater distress [14]. Patients could be categorized based on their individual sum scores: Non-case (0–7), borderline case (8–10) and definite case (11 and above) [14, 15]. To identify patients with at least moderate symptoms of anxiety and depression, we used a cut-off score of > 8 [11].

### Statistical analysis

Descriptive statistics were employed to categorise patients according to demographic, treatment-related and psychosocial characteristics. Student’s *t*-test was used to analyse group differences on the outcome variables at baseline. Analysis was performed with the following grouping variables (partially splitted by median values): sex, age (<59.6 years vs. ≥59.6 years), RPA classification, steroid uptake (yes or no), radiation (WBRT or hfSRT), family status (single vs. married) and educational level (low education vs. moderate education and vs. high education). In addition, we used repeated measures ANOVA to assess the effect of RT on distress, anxiety and depression. Next to the interaction effect of group (TG vs. CG) x time (baseline, 6 w, 3 mo, 6 mo post RT) we report the main effect ‘time’. A significant interaction effect would indicate that the time course of the self-perceived symptoms of distress, anxiety and/or depression differs across groups. In addition, repeated measures ANOVA was conducted with patients treated for BM using different RT schemes (WBRT vs. hfSRT) or surgery (yes vs. no) as the between-subject factor. The Cochran’s Q-test was used to detect a significant change over time in the percentage of patients having significant distress, anxiety and/or depression. Moreover, we report observed frequencies in the TG and the CG for each problem of the DT problem list.

Bivariate intercorrelations between DT scores and levels of anxiety and depression were determined by using the Pearson correlation coefficient. According to Cohen [16], a correlation coefficient of r = 0.10 is considered as small, of r = 0.30 as medium and of r = 0.5 as high (magnitude of effect size).

Statistical analyses were performed using IBM SPSS Statistics 20. The significance level was set to p ≤ 0.05. Alpha adjustment was not applied due to the exploratory purpose of this study.

## Results

### Study participants and drop outs

A total of 90 patients (TG) and 41 patients (CG) were eligible for participation. Reasons for non-response were, for instance, failure to return the questionnaires before RT (TG 12 patients and CG 1 patient). One patient from TG declined to take part (CG 8 patients). Four patients with cranial involvement had no RT although initially planned and three patients of TG had additional surgery during RT. Three patients with SCLC (small cell lung cancer) received a prophylactic cranial irradiation. A total of 67 patients of the TG (34 male and 33 female) and 32 women of the CG participated in the study.

Reasons for drop out after RT were failure to return questionaires even after a reminder (TG 19 patients, CG 8 patients) and deterioration of the general health condition in three patients of the TG. After 3 months, 10 patients with BM showed an intracranial progress; after 6 months 14 patients. Clinical follow up data was not available for 10 patients (TG) after 3 months and for 9 patients after 6 months. Twenty-eight patients of the TG died within six months of follow up (42%).

Seventeen patients of the TG and 22 patients of the CG answered the DT and problem list at all time points. Twenty-four of the TG and 22 of the CG always answered the HADS.

### Patient and treatment characteristics

In the TG, lung cancer was the most common primary (52.2%), followed by breast cancer (13.4%). WBRT was administered to 40 (59.7%) patients while hfSRT was employed in 27 (40.3%) patients. Seventeen patients (25.4%) had a neurosurgical resection of the lesions before RT, 10 of these followed by postoperative hfSRT. Patient and treatment characteristics are shown in Table 1.

Symptomatic patients received corticosteroid medication as required. A total of 40 patients of TG did not require corticosteroids before, during or up to 6 weeks after RT. The initial mean dexamethasone dose was 7.2 mg/d before RT, the maximal dose was 50 mg/d before RT and the minimal dose was 2 mg/d before RT.

### Baseline self-perceived distress, anxiety and depression

Before RT, the TG did not significantly differ from the CG with regard to HADS sum scores (*p*s ≥ 0.16). Both groups reported on average moderate anxiety while mean depression levels were low. However, evaluating the DT scores, the TG reported significantly higher distress than the CG (M = 5.6 vs. M = 4.6, *p* = 0.029) (see Table 2). Using a cut-off score of ≥5 70% of the TG and 66% of the CG suffered from relevant distress before RT (see Table 2).

**Table 2:** **Baseline scores on DT and HADS and classification**

	TG	CG	p value	TG				CG			
	Item score M ± SD (n)	Item score M ± SD (n)		cut-off ≥5				cut-off ≥5			
DT	5.55 ± 2.74 (50)	4.59 ± 1.78 (32)	0.029	n = 35 (70%)				n = 21 (66%)			
				Patient classes acc. Sum Score (non-, borderline-, definite case, at least moderate symptoms)				Patient classes acc. Sum Score (non-, borderline-, definite case, at least moderate symptoms)			
**HADS**	**Sum Score M ± SD (n)**	**Sum Score M ± SD (n)**		**0-7 (%)**	**8-10 (%)**	**≥ 11 (%)**	**> 8 (%)**	**0-7 (%)**	**8-10 (%)**	**≥ 11 (%)**	**> 8 (%)**
**HADS anxiety**	10.14 ± 3.93 (67)	9.06 ± 3.54 (32)	0.567	17 (25.4)	19 (28.4)	31 (46.3)	47 (70.1)	13 (40.6)	7 (21.9)	12 (37.5)	17 (53.1)
**HADS depression**	6.26 ± 4.52 (66)	3.90 ± 3.98 (32)	0.157	39 (58.2)	15 (22.4)	13 (19.4)	20 (30.3)	27 (84.4)	2 (6.3)	3 (9.4)	3 (9.4)

*Abbreviations*: CG = control group, DT = Distress Thermometer, HADS = Hospital Anxiety and Depression Scale, TG = treatment group.

n = numbers of patients; M = average; SD = standard deviation.

Analysis of baseline TG values on DT and HADS revealed a significant difference in global distress between patients intended to be treated with WBRT (n = 30) and hfSRT (n = 20). Patients intended to be treated with hfSRT (M = 6.60, SD = 2.56) experienced higher distress than patients intended to be treated with WBRT (M = 4.85, SD = 2.67, *p* = 0.025). In contrast, no differences on HADS scores occurred (*p*s ≥ 0.32). Age, sex, family status, education levels, work, RPA classes, steroid use and surgery had no significant influence on baseline scores.

### The course of distress, anxiety and depression depending on treatment

**Figure 1:**
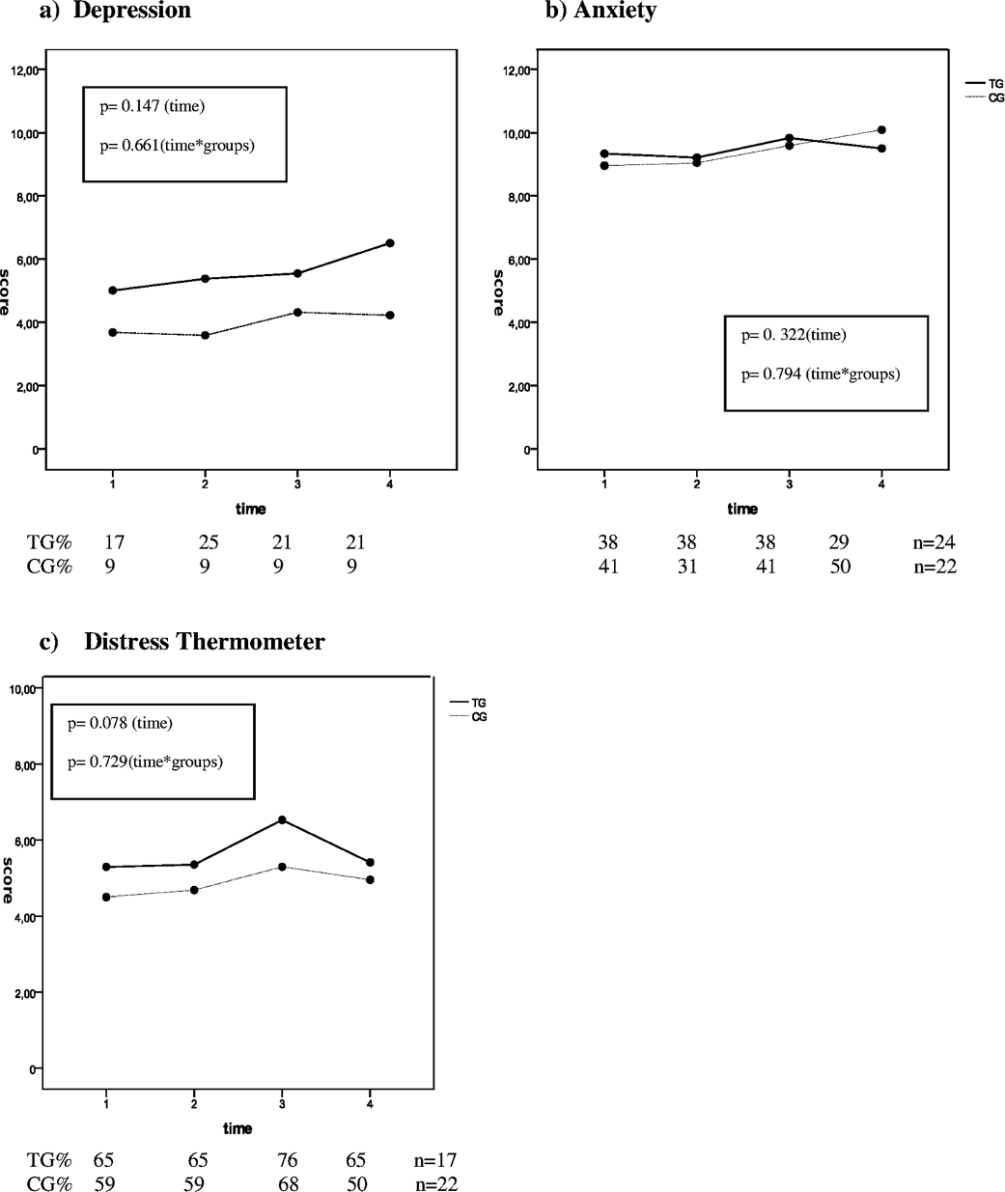
**The course of distress, anxiety and depression depending on radiotherapy treatment. a-c**: The course of distress, anxiety and depression depending on treatment time 1 = before RT, time 2 = 6 weeks after RT, time 3 = 3 months after RT, time 4 = 6 months after RT.

No significant interaction between time and group and no major effect of time on distress, anxiety and depression was observed (Figure 1a-c).

However, the depression score increased slightly in the TG from a mean score of 5.6 before RT to a mean score of 6.5 after 6 months (Figure 1a) and the distress values rose in the TG from a mean score of 5.3 before RT to a mean score of 6.5 after 3 months. After 6 months the distress level dropped to the baseline level (M = 5.4) (Figure 1c). Over the course of time no significant changes in the percentage of patients classified as significantly distressed (cut-off ≥ 5, TG: *p* = 0.343, CG: *p* = 0.29) or depressed (cut-off >8, TG: *p* = 0.494, CG: *p* = 0.392) were observed. In contrast, the percentage of patients in the TG with significant anxiety (cut-off >8) significantly changed over time (p = 0.035) while in the CG a trend towards significance was found (p = 0.054).

### The course of distress, anxiety and depression levels of TG patients depending on surgery and the radiotherapy protocol employed

Repeated measures ANOVA did not detect statistically significant group x time interactions. Thus, the time courses of anxiety and depression levels were similar for patients with (n = 8) and without surgery (n = 16) and patients with WBRT (n = 12) and hfSRT (n = 12), respectively (*p*s ≥ .06). In addition distress level was also similar for patients with (n = 7) and without surgery (n = 11) and patients with WBRT (n = 10) and hfSRT (n = 7, *p*s ≥ .06).

### Detailed analysis of the problem list included within the DT

Regarding the problem list, emotional and physical strains were most prevalent in both groups, but emotional stress featured more prominently than most of the physical problems. Before RT, 56% of the TG patients reported having concerns while 62% complained of having fears and 53% suffered from sadness. In the CG, 66% of the patients reported concerns 59% and 56% suffered from fear and sadness, respectively (Table 3).

**Table 3:** **Frequencies of reported problems (%)**

	TG				CG			
	T_0_	T_1_	T_2_	T_3_	T_0_	T_1_	T_2_	T_3_
	n = 67	n = 48	n = 34	n = 34	n = 32	n = 27	n = 26	n = 29
**Practical things**								
Housing	6	4	3	0	9	1	0	3
Insurance/ financial	6	2	3	0	9	7	4	7
Work/ school	2	5	3	9	3	7	8	10
Transportation	11	11	18	17	3	0	0	0
Childcare	2	2	4	0	0	4	4	3
**Family Problems**								
Significant others	8	6	12	9	1	22	20	14
Dealing with children	2	2	3	5	13	4	0	7
**Emotional**								
Concerns	56	49	59	59	66	56	58	52
Fears	62	54	53	78	59	41	50	55
Sadness	53	52	55	64	56	30	38	48
Depression	28	32	29	35	22	19	35	28
Nervousness	40	33	32	35	31	26	42	39
**Spirituality**								
God	5	2	6	4	3	3	0	0
Atheism	5	7	3	9	9	9	0	0
**Physical problems**								
Pain	41	46	31	35	44	41	58	62
Nausea	6	35	28	39	13	19	12	7
Fatigue	55	72	63	83	66	52	62	66
Sleep disorder	37	46	39	39	63	59	58	61
Movement	42	52	47	67	38	48	50	28
Washing/ Bathing	16	17	12	21	3	19	0	0
Appearance	18	23	15	21	22	19	8	10
Breathing	30	34	38	39	28	22	8	31
Mucositis	9	15	9	13	6	7	12	17
Eating	10	40	39	38	6	4	4	0
Digestion	22	27	18	29	19	7	8	17
Constipation	21	27	25	33	16	4	15	21
Diarrhea	11	15	9	17	13	19	4	7
Changes in urination	16	4	12	17	13	19	12	3
Fever	6	4	0	4	0	4	4	7
Itchy skin	20	44	24	52	38	19	27	38
Running nose	20	27	32	39	34	22	15	34
Tingling in hands/ feet	29	33	26	39	38	48	58	55
Edema	25	29	18	17	25	19	23	17
Sexual problems	23	25	26	48	16	22	23	18

*Abbreviations:* T_0_ = before RT, T_1_ = 6 weeks after RT, T_2_, T_3_ = 3, 6 months after RT.

### Bivariate intercorrelations of distress, anxiety and depression

To investigate the relation between DT scores and levels of anxiety and depression we analysed the correlation at each time point (Table 4). The correlation between DT and HADS scores was highest between DT and anxiety before RT (r = 0.565) and lowest between DT and depression 6 months after RT (r = 0.069).

**Table 4:** **Bivariate intercorrelations between DT and HADS scores**

	Time	HADS anxiety	HADS depression	n (TG/CG)	HADS anxiety correlated with HADS depression	n (TG/CG)
		Pearson correlation coefficient			Pearson correlation coefficient	
**DT**	before RT	0.565**	0.438**	82 (50/32)	0.711**	98 (66/32)
**DT**	6 weeks	0.379**	0.473**	64 (37/27)	0.578**	72 (45/27)
**DT**	3 months	0.334*	0.409*	54 (28/26)	0.760**	58 (32/26)
**DT**	6 months	0.197	0.069	50 (21/29)	0.445**	55 (26/29)

**The correlation is on the niveau to 0.01 significant.

*The correlation is on the niveau to 0.05 significant.

*Abbreviations:* DT = Distress Thermometer, HADS = Hospital Anxiety and Depression Scale.

HADS levels of anxiety and depression correlated significantly at all points of time, the highest correlation was found after 3 months (r = 0.760) and the lowest correlation was found 6 months after RT (r = 0.445).

## Discussion

In a palliative setting with a limited survival period, it is important to recognize if patients are suffering from anxiety, depression and distress in order to support them. In this study, we used two screening tools, namely the DT and the HADS to evaluate the course of psychological burden in patients with BM and RT to the brain in comparison to a CG of breast cancer patients. Both instruments were completed by the patients before RT, as well as 6 weeks, 3 months and 6 months after RT.

Before RT, both study groups had on average moderately high anxiety and low depression levels, while the scores of the DT were significantly higher in the TG. There were no significant between-group differences in the course of distress.

Goebel et al. [8] examined postoperative distress levels of patients with primary intracranial cancers. Using a cut-off score of ≥ 6 in the DT, 50% of their patients suffered from relevant distress. Using this higher cut-off score before RT in our study, we found similar results with 52% of the patients in the TG and 25% in the CG suffering from significant distress. Thus, the level of distress between patients with brain tumours and BM seem to be comparable. However it has to be taken into account that the operation of a primary intracranial cancer could also have an effect on the distress level.

Hinz et al. reported nearly twice as high levels of anxiety and depression in cancer patients compared to the general population [15]. In comparison to Hinz et al. (anxiety score, M = 7.2; depression score, M = 6.4) [15], we found even higher baseline anxiety levels in our groups (TG, M = 10.1; CG, M = 9.1) while depression levels were similar or lower (TG, M = 6.3; CG, M = 3.9). Thereby, Hinz et al. [15] assessed patients with different types of cancer like prostate and lung cancer as well as brain tumours. In the study conducted by Takahashi et al. [17], cancer patients answered the HADS before and after RT. Before RT, 15% of the patients suffered from anxiety or depression. Using the same cut-off (≥11), significant anxiety (46%) but not depression (19%) occurred more frequently in our TG at baseline. Our results therefore suggest that patients with BM undergoing RT to the brain suffer more often from symptoms of anxiety than cancer patients in general.

Although the TG had RT to the brain and a very limited survival time, the courses of their distress, anxiety and depression levels over time were similar to those observed in the CG. This suggests that the therapies were experienced as similarly distressing. In accordance to the study results of Goebel et al. [5], our patients suffered more often from emotional problems than physical ailments. Women with newly diagnosed breast cancer place emotional concerns above physical ailments, too [18], stressing the importance to deal with these symptoms.

Next to emotional problems, attention should be paid to the physical ailments. Like other authors [18], we found fatigue, pain and sleep disorders to be the most common physical problems. After RT, we found a high prevalence of nausea in the TG, but not in the CG. Nausea and vomiting are well-known side-effects of RT [19]. At 6 months following RT, nearly half of the TG complained about sexual problems, but only 18% of the CG. Sexual activity depends on the emotional situation; patients with depression have a decreased libido [20]. Regarding depression, sadness and fear, between 35-78% of the TG and 28-55% of the CG suffered from these terms at 6 months following RT which might explain the high frequency of sexual problems in the TG at this point in time. In contrast, sleep disorder and tingling in hands and feet occurred more often in the CG than in the TG. This might be due to hormonal treatment which influences the rhythm of sleep [21] and pre-treatment with chemotherapy which can cause nerve damages and prickling sensation/paraesthesia in the hands and feet [22].

Regarding the association between the DT and the HADS, we found a strong correlation (*r* = 0.57) between the baseline DT and HADS anxiety scores. Studies with patients after bone marrow transplantation (*r*_anxieties = 0.42, *r*_depression = 0.23) [23] or cancer patients before rehabilitation (r_anxieties = 0.45, r_depression = 0.39) [11] confirmed that distress is stronger correlated with anxiety than with depression. As most of the observed correlations were lower (*r* = 0.07 - 0.47), the two instruments used to screen for distress, anxiety and depression could not be replaced by each other. The HADS scales were more strongly associated. Petruzzi et al. [24] also found a high correlation between the two HADS scales in patients with brain cancer (*r* = 0.57). The results reveal that there is an overlap between anxiety and depression.

Our study has some limitations which have to be taken into account. In the palliative setting in which this study was conducted, the survival time of the patients in the TG was limited to a few months. The high drop-out rate over the study period resulted in a small sample size and limits the generalizability of our results. Moreover, this study could not detect later adjustment processes. Thus, future studies with survivors are important to analyse changes in anxiety, depression and distress in the long run. However, due to declines in the general state of health, high drop-out rates should be anticipated in this patient group. Because of ethical reasons, we decided to send only one reminder if a questionnaire was not answered by a patient.

In addition, study results should be interpreted with caution as breast cancer patients were chosen as CG. First, this is a gender-specific group. Second, breast cancer patients could have a hormonal dysfunction due to a therapy- induced menopause which can also influence emotional and physical concerns [25].

However, as far as we know, this is the first prospective longitudinal study on the course of distress, anxiety and depression in patients with BM. Despite the palliative setting and limited survival time of our TG, it was possible to implement tools to screen for distress, anxiety and depression in patients with BM.

## Conclusion

This exploratory study shows that patients with BM suffer from significantly higher global distress compared with breast cancer patients prior to RT while both groups showed high baseline anxiety levels and similar time courses of distress, anxiety and depression. Thus, patients with BM scheduled for RT should be early screened for global and specific distress. Patients with significant distress should be referred to a psycho-oncologist. Regarding physical problems, pain, sleep disorders and fatigue are the most prominent symptoms which should receive attention and supportive care, too. Furthermore, it can be concluded that both HADS and DT are practical and useful to identify distress, anxiety and depression in patients with BM and RT to the brain. Despite some overlap, they are not interchangeable as they measure different aspects of distress, especially after RT.

## Abbreviations

BM: Brain metastases
CG: Control group
CUP: Cancers of unknown primary
DT: Distress thermometer
HADS: Hospital anxiety and depression scale
hfSRT: Hypofractionated stereotactic radiotherapy
KPS: Karnofsky performance score
NCCN: National comprehensive cancer network
NCCN DT: National comprehensive cancer network- distress thermometer
RT: Radiotherapy
RPA: Recursive partitioning analysis
RTOG: Radiation therapy oncology group
SCLC: Small cell lung cancer
TG: Treatment group
WBRT: Whole-brain radiotherapy.
